# Resolution of fibromyalgia by controlling obstructive sleep apnea with a mandibular advancement device

**DOI:** 10.5935/1984-0063.20200077

**Published:** 2021

**Authors:** Fernanda Vantine, Dominik Ettlin, Miguel Meira-e-Cruz

**Affiliations:** 1 Universidade do Vale do Paraíba, Dental School - São José dos Campos - São Paulo - Brazil.; 2 University of Zurich, Center of Dental Medicine - Zurich - Zurich - Switzerland.; 3 Centro Cardiovascular da Universidade de Lisboa, Lisbon School of Medicine, Sleep Unit - Lisboa - Lisboa - Portugal.; 4 Faculdade São Leopoldo Mandic, Laboratory of Neuroimmune Interface of Pain Research - Campinas - São Paulo - Brazil.

**Keywords:** Mandibular Advancement, Sleep Apnea, Obstructive, Fibromyalgia

## Abstract

Fibromyalgia (FM) is a chronic, often disabling disorder characterized by multisite pain along with sleep problems and fatigue. Pain and sleep exhibit a reciprocal relationship. When FM and obstructive sleep apnea/hypopnea (OSA) co-exist, treatment options include continuous positive airway pressure or mandibular advancement device. We present a patient experiencing fibromyalgia and OSA whose symptoms vanished wearing a Mandibular Advancement Device (MAD) during sleep. To our knowledge, this is the first documented case of FM symptom resolution by MAD treatment.

## INTRODUCTION

According to the latest expert definition, a diagnosis of fibromyalgia (FM) is established when a patient experiences multisite pain defined as 6 or more pain sites from a total of 9 possible sites, and moderate to severe sleep problems and/or fatigue for at least 3 months^1^.

Additionally, a high proportion of patients with FM report relevant restrictions of daily activities (65%), depression (34%) and anxiety (25%)^2^. Poor sleep is reported by almost 80% of patients with FM^3^. In fact, epidemiological data suggest poor sleep quality to be a dose-dependent risk factor for fibromyalgia^4^; vice versa, self-reported restorative sleep was independently associated with the resolution of chronic widespread pain^5^. In general, chronic pain has been associated with sleep disturbances in a bidirectional manner with pain disrupting sleep and sleep deprivation or disturbance increasing pain^6^. A meta-analysis of studies using polysomnography (PSG) revealed that individuals with FM compared to healthy controls had longer duration of wakefulness during sleep, shorter sleep duration, lower sleep efficiency, spent more time in light sleep (i.e., a higher percentage of stage 1 sleep and a lower percentage of slow wave sleep [SWS]). Although several studies reported a comorbidity of FM and obstructive sleep apnea/hypopnea (OSAH), it is unknown what proportion of patients with FM also experience OSA^7-9^.^ ^ In patients with FM and OSAH, no correlation was observed between the degree of sleep disorder and severity of pain, pain duration, disability, or quality of life^7,8^. Only when OSAH and insomnia co-occur, significantly higher pain levels were observed in patients with FM^10^.

Continuous positive airway pressure (CPAP) is the first-line treatment for patients with moderate to severe OSAH, ameliorating respiratory distress, improving daytime sleepiness, quality of life, blood pressure levels, and cognition^11^. Treatment with nasal CPAP resulted in an improvement in functional symptoms as assessed by a validated questionnaire^11^. However, despite the high efficacy of this device, CPAP adherence is often sub-optimal^12,13^. Mandibular advancement devices (MADs) increasingly become an effective treatment alternative for OSAH^14^. MADs reduce the apnea/hypopnea index (AHI), sleep arousals, and daytime fatigue. Further, they improve oxygen saturation as well as quality of life^15^. Finally, MADs reduce the blood pressure significantly and to a similar extend as CPAP^16^. Despite greater efficacy of CPAP in reducing the AHI, studies revealed comparable health outcomes with CPAP and MAD treatment^15,16^. Here, we present a patient with fibromyalgia and OSA whose symptoms resolved by MAD treatment.

### History

A 61-year-old female was originally referred to a rheumatologist for evaluation and treatment of FM that started at age 40. Her quality of life was poor due to FM. She had no other disease or family history for any type of chronic pain. Her complaint was multisite body pain (joints of the hands, wrists, elbows, shoulders, and knees) with functional limitations such as covering herself at night with a blanket or independently climbing stairs. For pain control, the patient was prescribed daily doses of duloxetine 60mg (antidepressant), carbamezepine 200mg (anticonvulsant) and cyclobenzaprine hydrochloride 5mg (myorelaxant) that leveled her pain at 6/10 on a numeric rating scale (NR). After menopause around age 50, she developed a panic disorder and worsening of symptoms to 8/10 on the VAS. Besides experiencing chronic bodily pain for decades, she also reported frequent headaches (chronic migrain type, every day with intensity of 6/10), snoring, fragmented sleep, and excessive daytime sleepiness. The rheumatologist therefore requested a PSG evaluation.

### Polysomnography

The type 1 PSG was obtained by the brain wave III PSG device in a specialized sleep laboratory scored by a polysomnography technician and reviewed by a sleep specialist. Respiratory events were scored using the American Academy of Sleep Medicine scoring manual (version 2.4)^17^ and revealed a severe OSAH with a characteristic alfa-delta pattern typically found in FM patients.

### Self-report instruments

The patient completed four self-report instruments before and after treatment:

A numeric rating scale (NR) is an 11-point scale to measure pain intensity with the anchors no pain (0/10) and worst pain imaginable (10/10). It allows repeated accurate pain measurements^18,19^.

The Epworth sleepiness scale (ESS) is a self-administered questionnaire with 8 questions. Respondents are asked to rate, on a 4-point scale (0-3), their usual chances of dozing off or falling asleep while engaged in eight different activities. Scores reflect a person’s average sleep propensity in daily life (ASP), or their ‘daytime sleepiness’^20,21^.

The revised form of fibromyalgia impact questionnaire (FIQR) is an instrument developed to assess the current health status of women suffering from fibromyalgia. It has been applied in clinical and research settings. The 2009 FIQR version, which was used here, consists of 21 items across the 3 domains of function, overall impact, and symptoms. The maximum score is 100 indicating the worst health status^22,23^.

Patient health questionnaire 9 (PHQ-9): the PHQ-9 assesses severity of depression. Summary scores range from 0 to 27, indicating depression levels of “none/minimal” (0-4), “mild” (5-9), “moderate” (10-14), “moderately severe” (15-19), or “severe” (>19). A cut-off score range of 8-11 has been recommended for expert evaluation referral^24,25^.

### Physical exam

The patient had an increased body mass index of 34.37kg/m^2^. The physical exam revealed a normal nasal breathing pattern and good nasal patency. There mandible was freely mobile without indication of a temporomandibular joint abnormality. Oral and dental exams revealed good oral hygiene, a normal dental occlusion (angle class I), a normal-sized tongue (Mallampati class II) and readily visible tonsils (Friedman I palatal position).

### Treatment

The recommended therapy was continuous positive airway pressure (CPAP). but was not tolerated by the patient who felt very uncomfortable. Therefore, she was referred for treatment with a MAD. The mandibular advancement was determined by a double component, titratable device PM Positioner^®^ device with an initial set to 50% of maximum protrusion (8 of 16mm) with additional 2mm (1mm per appointment) and a final subjectively titrated setting of 10mm (62,5% of maximal protrusion).

### Outcome

After initiating treatment with the subjectively titrated MAD, the patient returned for follow - up visits after 3, 6 and 12 months. She continuously used the MAD every night and all night long confirmed by sleep diary including adherence related information. Self-reported control of snoring was confirmed by her husband and the Snorelab^®^ cell phone app. Improvement of excessive daytime sleepiness was observed soon after treatment initiation with the MAD without any negative side- effects. Her multisite pain resolved completely without further need for either previously prescribed analgesic medication and her mood improved much. The alfa-delta pattern was absent at this time. The scores of self-report instruments and PSG findings before and after 12 months of MAD treatment are presented in Tables 1 and 2.

**Table 1.** Scores of self-report instruments before and 6 months after treatment with a MAD.

Self-report instruments	Maximum index	Before MAD Before MAD	After MAD treatment	Normal values (cut-off score)
NR	10	8	0	0
ESS	24	20	3	9
FIQR	100	78.7	8.1	0
PHQ-9	27	26	2	4

NR: scale; ESS: Epworth sleepiness scale; FIQ: Fibromyalgia impact questionnaire revised; PHQ-9: Patient health questionnaire-9.

**Table 2.** PSG findings.

	TRT	TST	%N1	%N2	%N3	%REM	RDI	AHI	REM AHI	AI	HI	T90
Baseline PSG	498.0 min	334.5	4.0	70.3	11.1	14.6	43.0	69,8	38,5	10,8	38.5	0.7 min
Post- Treatment PSG	476.5 min	358	4.2	59.8	19	17	4.4	12,6	2,6	0,7	3.7	0.5 min

TRT: Total recording time; TST: Total sleep time; %N1: Percentage of stage N1 sleep; %N2: Percentage of stage N2 sleep; %N3: Percentage of stage N3 sleep; %REM: Percentage of stage REM sleep, AHI: AHI; AI: Apnea index; HI: Hypopnea index; T90: Maximum time with oxygen saturation below 90%.

## DISCUSSION

Due to intolerance of CPAP treatment, this patient opted for a MAD as the primary intention to treat her OSAH. Yet unexpectedly, her chronic multisite pain also resolved. To our knowledge, this is the first report of a patient experiencing symptom relieve of both FM and severe OSAH with a MAD. The following considerations address possible mechanisms for this clinical observation.

Among other factors, pain amplification is thought to be related to an imbalance of neurotransmitters in the central nervous system (CNS). Chronic pain is associated with a dysregulation of the analgesic neurotransmitter serotonin, as well as an increase of the pain mediator substance P. Abnormalities in serotonin metabolism are also relevant in FM and depression^26^. Serotonin is also involved in the respiratory control at multiple sites either in the CNS and peripheral nervous system (PNS)^27^. OSA severity has been demonstrated to improve with intake of serotonin reuptake inhibitors^28^. EEG studies on sleep in individuals with FM revealed a pattern characterized by intrusion of alpha waves during stage 4 of non-REM sleep. This pattern is referred to by patients as a waking state during sleep, or as non-restorative and superficial sleep, during which frequent arousals occur, commonly triggered by weak stimuli. These EEG changes were associated with fatigue and generalized pain^29^. Deprivation of the deep phases of non-REM sleep in normal volunteers can lead to morning fatigue and fibromyalgia manifestations^3^. Headaches may be intrinsically related to sleep, may cause sleep disturbances, or manifestations of sleep apnea. Nevertheless, sleep disorders may still be associated with other primary headaches, especially tension-type headaches (TTH)^30^ and morning headaches with migraine-like features^31^. Migraineurs with excessive daytime sleepiness (EDS) experienced more severe headache intensity, reported a higher impact of the headache, and more depressive symptomology than those without EDS. These findings suggest that migraineurs with EDS are more burdened than migraineurs without EDS^32^. Also this patient reported an improvement of her headaches, presenting in the post therapeutic assessment with occasional pain (once each two or three months), as well as documented decreased ESS scores.

Evidence implicates CNS and sleep disorders (difficulty falling asleep, difficulty staying asleep and early morning awakening) as keys to perpetuating pain and other core symptoms of FM and related conditions. An inverse correlation was demonstrated between sleep quality and pain threshold in subjects with FM^11^. Findings from animal studies (rats) revealed a decreased pain sensitivity in response to chronic intermittent hypoxia, which is possibly due to increased activation of the hypoxia-inducible factor (HIF)-1α and increased opioid receptors^33^. If OSAH indeed contributes to heightened pain levels, treating the underlying sleep apnea could decrease pain perception, and eventually lead to decrease use of analgesic medication^18^. Although the successful treatment of the severe OSAH and consequently the resolution of related intermittent hypoxia may explain the lack of need for analgesics (as no more pain was experienced), this could be also attributable to the reduction in the arousal index subsequent to OSA treatment^34^.

Patients experiencing a panic disorder commonly suffer from insomnia (approximately 70% of patients) and fragmented, non-restorative sleep^36^. The incidence of OSAS in women increases after menopause indicating that female sex hormones may have a protective effect^37^. Hormones may influence the neuronal ventilatory control, the mechanical behavior of the upper airways, or patterns of body fat distribution^38^. The administration of hormones (progesterone and estrogen) to postmenopausal men or women reduces the AHI, which seems to confirm the effect of sex hormones in the etiopathogenesis of OSAS^39^. Interestingly, sex hormones are critical mediators regarding the relationship between slow wave sleep loss and cardiometabolic risk^40^, which may explain the increased prevalence of both these features in FM patients^41^.

Another case of severe OSAS and FMS symptoms with total symptom resolution with nasal CPAP treatment has been published^42^. In our report, due to the intolerance of CPAP, we opted for the use of a MAD that effectively controlled the symptoms of severe OSAH and FM. To our knowledge, this is the first documented case in which comorbid OSAH and FM were successfully controlled with a MAD.

Larger studies are necessary to confirm our findings and to better understand the efficacy and safety of MADs in patients with FM and severe OSAH.

## Figures and Tables

**Figure 1:**
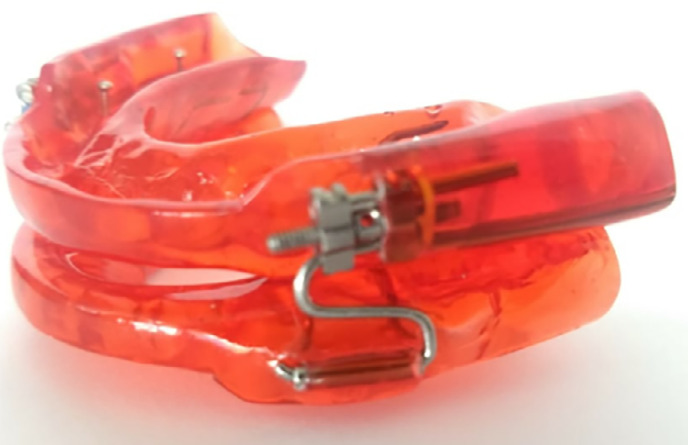
Mandibular Advancement Device.

**Figure 2:**
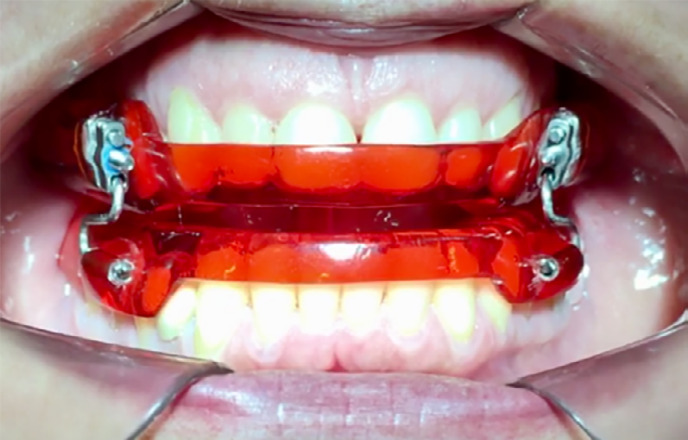
Mandibular Advancement Device in situ

## References

[1] Arnold L, Bennett RM, Crofford LJ, Dean LE, Clauw DJ, Goldenberg DL (2019). AAPT diagnostic criteria for fibromyalgia. J Pain.

[2] Gustorff B, Dorner T, Likar R, Gridold W, Lawrence K, Schwarz F (2008). Prevalence of self-reported neuropathic pain and impact on quality of life: a prospective representative survey. Acta Anaesthesiol Scand.

[3] Wu YL, Chang LY, Lee HC, Fang SC, Tsai PS (2017). Sleep disturbances in fibromyalgia: a meta-analysis of case-control studies. J Psychosom Res.

[4] Mork PJ, Nilsen TIL (2012). Sleep problems and risk of fibromyalgia: longitudinal data on an adult female population in Norway. Arthritis Rheum.

[5] Davies K, Macfarlane GF, Nicholl BI, Dickens C, Morriss R, Ray D (2008). Restorative sleep predicts the resolution of chronic widespread pain: results from the EPIFUND study. Rheumatology (Oxford).

[6] Andersen ML, Araujo P, Frange C, Tufik S (2018). Sleep disturbance and pain: a tale of two common problems. Chest.

[7] Meresh ES, Artin H, Joyce C, Birch C, Daniels D, Owens JH (2019). Obstructive sleep apnea co-morbidity in patients with fibromyalgia: a single-center retrospective analysis and literature review. Open Access Rheumatol.

[8] Aytekin E, Demir SE, Komut EA, Okur SC, Burnaz O, Caglar NS (2015). Chronic widespread musculoskeletal pain in patients with obstructive sleep apnea syndrome and the relationship between sleep disorder and pain level, quality of life, and disability. J Phys Ther Sci.

[9] May KP, West SG, Baker MR, Everett DW (1993). Sleep apnea in male patients with the fibromyalgia syndrome. Am J Med.

[10] Mundt JM, Eisenschenk S, Robinson ME (2018). An examination of pain's relationship to sleep fragmentation and disordered breathing across common sleep disorders. Pain Med.

[11] Marvisi M, Balzarini L, Mancini C, Ramponi S, Marvisi C (2015). Fibromyalgia is frequent in obstructive sleep apnea and responds to CPAP therapy. Eur J Intern Med.

[12] Mehrtash M, Bakker JP, Ayas N (2019). Predictors of continuous positive airway pressure adherence in patients with obstructive sleep apnea. Lung.

[13] Canadian Agency for Drugs and Technologies in Health (2013). CPAP treatment for adults with obstructive sleep apnea: review of the clinical and cost-effectiveness and guidelines.

[14] Dieltjens M, Vanderveken O (2019). Oral appliances in obstructive sleep apnea. Healthcare (Basel).

[15] Kuhn E, Schwarz EI, Bratton DJ, Rossi VA, Kohler M (2017). Effects of CPAP and mandibular advancement devices on health-related quality of life in OSA: a systematic review and meta-analysis. Chest.

[16] Bratton DJ, Gaisl T, Wons AM, Kohler M (2015). CPAP vs mandibular advancement devices and blood pressure in patients with obstructive sleep apnea: a systematic review and meta-analysis. JAMA.

[17] Berry RB, Brooks R, Gamaldo C, Harding SM, Lloyd RM, Quan SF (2017). AASM scoring manual updates for 2017 (Version 2 4). J Clin Sleep Med.

[18] Langley GB, Sheppeard H (1985). The visual analogue scale: Its use in pain measurement. Rheumatol Int.

[19] Hawker GA, Mian S, Kendzerska T, French M (2011). Measures of adult pain: Visual Analog Scale for Pain (VAS Pain), Numeric Rating Scale for Pain (NRS Pain), McGill Pain Questionnaire (MPQ), Short-Form McGill Pain Questionnaire (SF-MPQ), Chronic Pain Grade Scale (CPGS), Short Form-36 Bodily Pain Scale (SF-36 BPS), and Measure of Intermittent and Constant Osteoarthritis Pain (ICOAP). Arthritis Care Res.

[20] Johns MW (1991). A new method for measuring daytime sleepiness: the Epworth sleepiness scale. Sleep.

[21] Bertolazi AN, Fagondes SC, Hoff LS, Pedro VD, Barreto SSM, Johns MW (2009). Portuguese-language version of the Epworth sleepiness scale: validation for use in Brazil. J Bras Pneumol.

[22] Bennett RM, Friend R, Jones KD, Ward R, Han BK, Ross RL (2009). The revised fibromyalgia impact questionnaire (FIQR): validation and psychometric properties. Arthritis Res Ther.

[23] Marques AP, Santos AMB, Assumpção A, Matsutani LA, Lage LV, Pereira CAB (2006). Validação da versão brasileira do fibromyalgia impact questionnaire (FIQ). Rev Bras Reumatol.

[24] Kroenke K, Spitzer RL, Williams JB (2001). The PHQ-9: validity of a brief depression severity measure. J Gen Intern Med.

[25] Santos IS, Tavares BF, Munhoz TN, Almeida LSP, Silva NTB, Tams BD (2013). Sensibilidade e especificidade do patient health questionnaire-9 (PHQ-9) entre adultos da população geral. Cad Saúde Pública.

[26] Schwarz MJ, Offenbaecher M, Neumeister A, Ewert T, Willeit M, Praschak-Rieder N (2002). Evidence for an altered tryptophan metabolism in fibromyalgia. Neurobiol Dis.

[27] Hilaire G, Voituron N, Menuet C, Ichiyama RM, Subramanian HH, Dutschmann (2010). The role of serotonin in respiratory function and dysfunction. Respir Physiol Neurobiol.

[28] Cheng JY (2018). Serotonin reuptake inhibitors in obstructive sleep apnea: associations in people with and without epilepsy. Neurol Res Int.

[29] Moldofsky H (2008). The significance of the sleeping-waking brain for the understanding of widespread musculoskeletal pain and fatigue in fibromyalgia and allied syndromes. Joint Bone Spine.

[30] Chiu YC, Hu HY, Lee FP, Huang HM (2015). Tension-type headache associated with obstructive sleep apnea: a nationwide population-based study. J Headache Pain.

[31] Suzuki K, Miyamoto M, Miyamoto T, Numao A, Suzuki S, Sakuta H (2015). Sleep apnoea headache in obstructive sleep apnoea syndrome patients presenting with morning headache: comparison of the ICHD-2 and ICHD-3 beta criteria. J Headache Pain.

[32] Kim J, Cho SJ, Kim WJ, Yang KI, Yun CH, Chu MK (2016). Excessive daytime sleepiness is associated with an exacerbation of migraine: a population-based study. J Headache Pain.

[33] Wu J, Li P, Wu X, Chen W (2015). Chronic intermittent hypoxia decreases pain sensitivity and increases the expression of HIF1a and opioid receptors in experimental rats. Sleep Breath.

[34] Smith M, Perlis ML, Smith MS, Giles DE, Carmody TP (2000). Sleep quality and presleep arousal in chronic pain. J Behav Med.

[35] Meira e Cruz M, Manetta IP (2019). Sleep and pain: a circadian multi-challenge rather than a simple bidirectional pathway. Br J Pain.

[36] Lucchesi LM, Pradella-Hallinan M, Lucchesi M, Moraes WAS (2005). Sleep in psychiatric disorders. Rev Bras Psiquiatr.

[37] Robinson TD, Grundstein RR, Eckel RH (2003). Obesity - mechanisms and clinical management.

[38] Brooks LJ, Strohl KP (1992). Size and mechanical properties of the pharynx in health men and women. Am Rev Respir Dis.

[39] Krystal A, Edinger J, Wohlgemuth W, Marsh GR (1998). Sleep in peri-menopausal and post-menopausal women. Sleep Med Rev.

[40] Meira e Cruz M, Gozal D (2020). Slow-wave sleep loss and cardiometabolic dysfunction: androgenic hormone secretion as a critical intermediate mediator. Sleep Med.

[41] Su CH, Chen JH, Lan JL, Wang YC, Tseng CH, Hsu CY (2015). increased risk of coronary heart disease in patients with primary fibromyalgia and those with concomitant comorbidity - a Taiwanese population-based cohort study. PLoS One.

[42] Sepici V, Tosun A, Köktürk O (2007). Obstructive sleep apnea syndrome as an uncommon cause of fibromyalgia: a case report. Rheumatol Int.

